# Nutrient synergy: definition, evidence, and future directions

**DOI:** 10.3389/fnut.2023.1279925

**Published:** 2023-10-12

**Authors:** Jeremy R. Townsend, Trevor O. Kirby, Philip A. Sapp, Adam M. Gonzalez, Tess M. Marshall, Ralph Esposito

**Affiliations:** ^1^Research, Nutrition, and Innovation, Athletic Greens International, Carson City, NV, United States; ^2^Health & Human Performance, Concordia University Chicago, River Forest, IL, United States; ^3^Department of Allied Health and Kinesiology, Hofstra University, Hempstead, NY, United States; ^4^Department of Nutrition, Food Studies, and Public Health, New York University-Steinhardt, New York, NY, United States

**Keywords:** synergy, potentiation, phytonutrients, micronutrients, minerals, vitamins, polyphenols, supplementation

## Abstract

Nutrient synergy refers to the concept that the combined effects of two or more nutrients working together have a greater physiological impact on the body than when each nutrient is consumed individually. While nutrition science traditionally focuses on isolating single nutrients to study their effects, it is recognized that nutrients interact in complex ways, and their combined consumption can lead to additive effects. Additionally, the Dietary Reference Intakes (DRIs) provide guidelines to prevent nutrient deficiencies and excessive intake but are not designed to assess the potential synergistic effects of consuming nutrients together. Even the term synergy is often applied in different manners depending on the scientific discipline. Considering these issues, the aim of this narrative review is to investigate the potential health benefits of consuming different nutrients and nutrient supplements in combination, a concept we define as nutrient synergy, which has gained considerable attention for its impact on overall well-being. We will examine how nutrient synergy affects major bodily systems, influencing systemic health. Additionally, we will address the challenges associated with promoting and conducting research on this topic, while proposing potential solutions to enhance the quality and quantity of scientific literature on nutrient synergy.

## Introduction

Nutrient synergy refers to the concept that the combination of two or more nutrients working in conjunction exert greater physiological impact on the body than each nutrient consumed in isolation (1–5) — in other words, the whole is greater than the sum of the parts. Traditionally, nutrition science has utilized the reductionist approach to understand the impact of nutrition, diet, and nutrients on health, where most research attempts to draw conclusions by isolating a single nutrient and examining its effect on a specific health outcome or biological system (6–9). The study of how a single nutrient influences the body is of course not without merit, as we know that the consumption of single nutrients in appropriate amounts contributes to the prevention of certain nutrient deficiencies (10–13). This is classically demonstrated with the consumption of vitamin D to prevent rickets, vitamin C to prevent scurvy, or folic acid to prevent neural tube defects (14). As such, the Dietary References Intakes (DRIs) were established by the Food and Nutrition Board of the National Academies of Sciences Engineering, and Medicine and consist of several types of nutrient reference values, which are intended to reduce the risks of both nutrient inadequacy and excessive nutrient intake (15, 16). However, one shortcoming of the DRI framework is that it does not account for the additive effect some nutrients possess when consumed concurrently. Fortunately, during the Food and Nutrition Board’s recent discussions in 2021 on an update to Riboflavin guidelines, the board acknowledged the need to examine evidence for nutrient “clusters” for all DRI nutrients, considering these nutrients are not consumed in isolation and have metabolic interactions (16).

In pharmacology, synergy is discussed in various ways, such as through the enhancement of drug absorption, distribution, metabolism, or elimination (ADME). Changes in ADME result from interactions between drugs that affect different biological targets in the body, such as enzymes, receptors, or ion channels (17, 18). In other research disciplines, synergy has been interpreted more broadly in that ingredients that target differing physiological pathways work together for a greater physiological impact. For example, one ingredient may provide an energy substrate to improve physical capacity directly, while another nutrient may decrease fatigue through a separate pathway (19, 20). Furthermore, a recent meta-analysis of 35 randomized controlled trials found significantly greater gains in fat-free mass and strength in healthy adults consuming multi-ingredient supplements, that influence multiple physiological endpoints, compared to those consuming protein alone (21).

**Figure 1:**
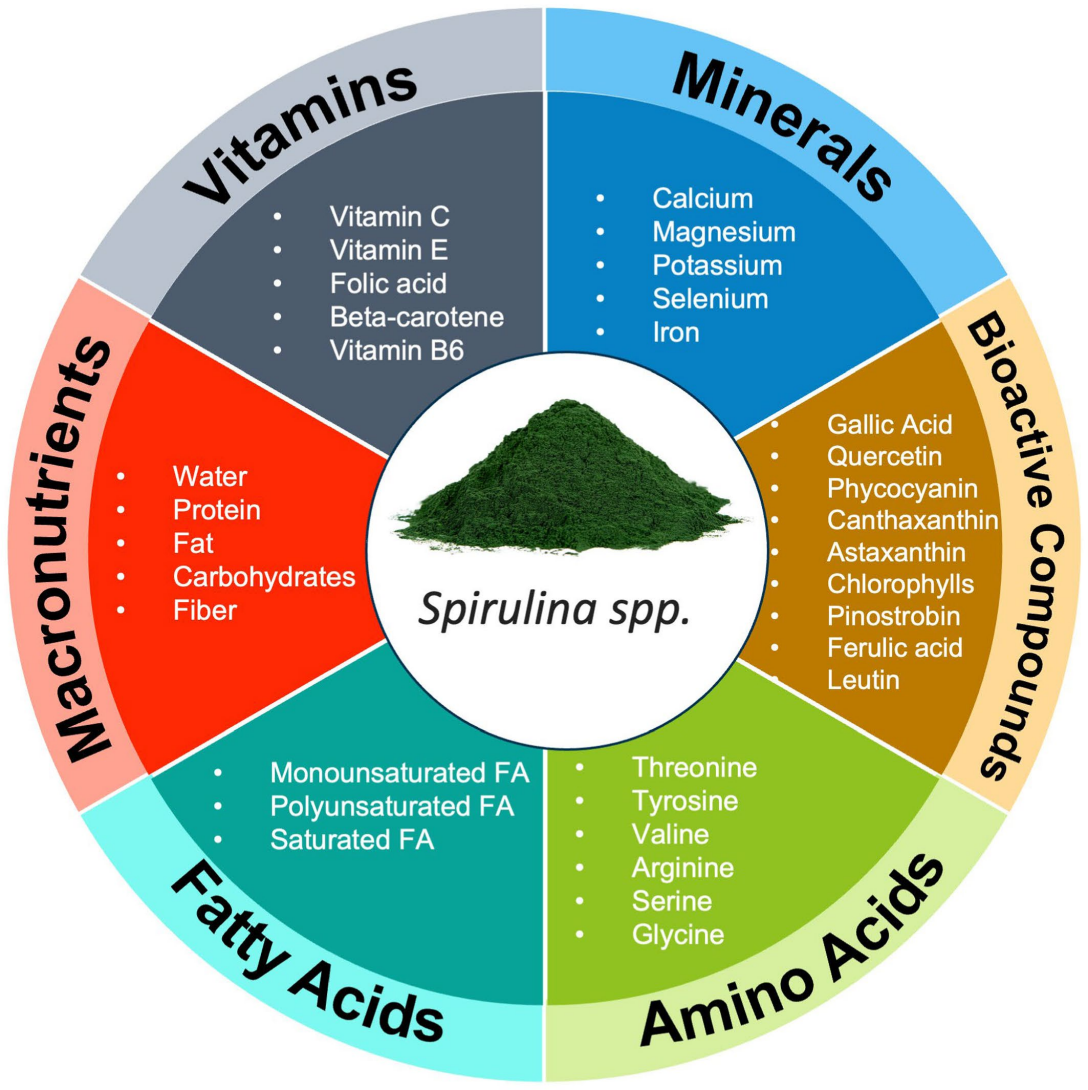
Example of Nutrient Synergy in foods (Spirulina spp.). This figure illustrates the diverse array of nutrients found in foods, highlighting their vital roles in supporting human health and well-being. In addition to macronutrients, foods provide an intricate blend of micronutrients, phytonutrients, and bioactive compounds, each contributing to various physiological functions. FA, fatty acid.

Nutrient synergy is commonly discussed in nutrition with regards to the way nature provides a multitude of nutrients in whole food sources (1–5). To date, over 10,000 different phytonutrients have been discovered with many likely still unidentified (22). Fruits, vegetables, legumes, and nuts contain a complex matrix of phytonutrients which are orchestrated to produce positive biological effects on the human body (Figure 1). Further, the concept of “eating the rainbow” has gained popularity in recent years as practitioners and researchers acknowledge the benefits of consuming various “color groups” of foods due to the unique blend of phytonutrients associated with the color of a plant (23–25). This strategy is reinforced by data indicating an inverse relationship between fruit and vegetable intake and the reduction of all-cause mortality and specifically cancer, depression, cardiovascular disease, and respiratory disease (26–28).

For this discussion, we define nutrient synergy as the dynamic interaction between different nutrients in the body, where their combined effects are greater than the sum of their individual contributions. The purpose of this review is to explore the health benefits of consuming various nutrients and nutrient supplements in combination. This phenomenon has garnered significant attention in the context of human health, and we will examine the effects of nutrient synergy on major bodily systems. Furthermore, we will discuss the inherent challenges when promoting and conducting research on nutrient synergy. Lastly, we hope to provide possible solutions to increase the quality and quantity of scientific literature on nutrient synergy, including proposing innovative research methodologies to explore this concept.

### Nutrient synergy and nervous system health

Nutrient synergy can improve brain health by enhancing cognitive function, supporting neuroprotective mechanisms, and regulating depression or anxiety as certain combinations of nutrients work synergistically to promote optimal brain function and reduce the risk of neurodegenerative diseases. In particular, human trials have demonstrated benefit from supplementing with a combination of B vitamins (e.g., vitamin B12, folate, and vitamin B6) on homocysteine levels and other aspects of nervous system health. Analysis of the large VITATOPS study, which was a cohort of 1,400 participants expanding over 10 countries, found a significant reduction (~4 μmol/L) in homocysteine in the group receiving vitamin B12, folate, and vitamin B6 supplementation compared to the control (29, 30). Researchers also found that the B vitamin treated group experienced a slowing of brain white matter loss progression, which may be attributed to the reduction in homocysteine (30). The mechanism is not entirely clear but it is hypothesized that the reduction in homocysteine from B vitamin supplementation may exert favorable and protective effects on nerve demyelination (31), and may attenuate the neurotoxic effects of homocysteine and N-methyl-D-aspartate (NMDA) agonists (32). Additionally, a randomized double-blind placebo controlled trial combining coenzyme Q 10 (CoQ10) with a multivitamin blend found that this combination may modulate parameters involved in blood flow to the brain which the researchers hypothesized provide a beneficial effect on neurovascular function (33).

Regarding anxiety and stress, Boyle et al. (34) investigated the effects of nutrient synergy on stress and anxiety in 100 moderately stressed adults. Participants received oral supplementation of either (1) Rhodiola + green tea + magnesium (Mg) + B vitamins; (2) Rhodiola + Mg + B vitamins; (3) Green tea + Mg + B vitamins; or (4) placebo in a double-blind parallel design before being exposed to the Trier Social Stress Test which involved delivering a speech and mathematical cognitive test in front of an unresponsive human panel. A synergistic effect was observed as most interventions provided some positive effects, but the most profound increase in electroencephalogram (EEG) resting state theta activity (indicative of a relaxed and alert state) was observed in the group where all the nutrients were provided in one treatment (Rhodiola + green tea + Mg + B vitamins). Furthermore, the blend of all ingredients attenuated subjective stress, anxiety, and mood disturbance, and heightened subjective and autonomic arousal to the greatest extent (Table 1). A follow-up study from this lab demonstrated the same combination of ingredients (Rhodiola + green tea +Mg + B vitamins) increased spectral theta brain activity while performing two attentional tasks suggesting an increased attentional capacity under conditions of stress compared to smaller isolated groups of ingredients (54). The synergistic effects of Mg and vitamin B6 were also examined as a therapy for anxiety-related premenstrual symptoms (55). Forty-four adult women were randomly assigned to one of four groups: (1) 200 mg Mg; (2) 50 mg vitamin B6; (3) 200 mg Mg + 50 mg vitamin B6; or (4) placebo. After supplementation through one full menstrual cycle, the investigators reported a synergistic effect of the combined Mg + vitamin B6 supplementation for reducing premenstrual anxiety related symptoms to a greater extent than either ingredient alone. Another study investigating the effects of nutrient synergy on Alzheimer’s disease severity provided participants with either a placebo, omega-3 fatty acids alone [675 mg docosahexaenoic acid (DHA) and 975 eicosatetraenoic acid (EPA)], or omega-3 fatty acids + alpha-lipoic acid (ɑLA; 600 mg) taken daily for 1 year (35). Data revealed that there was significantly less decline in the Mini-Mental State Examination score and Activities of Daily Living assessment for the omega-3 fatty acid + ɑLA group only. Based on the results, the authors concluded that the combination of ɑLA with omega-3 fatty acids was effective at slowing the cognitive and functional decline in Alzheimer’s disease over a 12-month period.

**Table 1:** Examples of nutrient synergy benefits in clinical trials.

Body system	Synergistic ingredients	Outcome
Nervous	Rhodiola + Magnesium + Green Tea + B-vitamins ^S^	Attenuated stress, heightened subjective and autonomic arousal, increased EEG theta activity (34)
	Vitamin B12, Folate, and Vitamin B6 ^C^	Reduction in homocysteine; Slowing of brain matter loss progression (29, 30)
	Omega-3 + Alpha Lipoic Acid ^S^	Less decline in the Mini-Mental State Examination score and activities of daily living assessment (35)
Cardiovascular	Folic Acid + Vitamin B12 ^S^	Reductions in homocysteine (36)
	Coenzyme Q10 + Vitamin E ^S^	Reduced low-density lipoprotein cholesterol (LDL-C), increased high-density lipoprotein cholesterol (HDL-C), reduced atherogenic coefficient (37)
	Omega-3 + Niacin ^S^	Increased LDL apoE/apoB ratios and LDL apoA1/apoB ratios (38)
Respiratory	Vitamin C + Vitamin E ^C^	Protection against the acute effects of ozone pollution (39)
	Vitamin C + Vitamin E + Beta-carotene ^C^	Improved FVC, FEV, and forced expiratory flow in hazardous environmental conditions (40)
Digestive	Synbiotic (probiotic + prebiotic)^S^	Greater quality-of-life improvements and a significant decrease in CRP levels (41)
	Synbiotic (probiotic + prebiotic) ^C^	More effective in eradicating *Helicobacter pylori* when combined with a standard medication therapy (42)
Endocrine	Zinc + Selenium ^S^	Improved T3, free T4, and TSH levels (43)
	Vitamin D + Calcium + Leucine-enriched whey protein drink ^C^	Suppression of parathyroid hormone, increased serum 25(OH)D, and accompanying small improvements in bone mineral density (44)
Musculoskeletal	Calcium + Vitamins D + Vitamin K ^S^	Improved bone mineral density, circulating levels of biomarkers associated with bone health, and risks of fractures (45, 46)
	Vitamin D + Calcium ^S^	Reduced parathyroid hormone and increased calcium/creatinine ratio (47)
Immune	*Chlorella vulgaris* + Vitamin E ^S^	Attenuation of TNF-*α* levels in patients with NAFLD (48)
	Vitamin C + Zinc ^C^	Symptom relief from the common cold (49)
	Vitamin C + Vitamin E ^S^	Enhanced immune response (50)
Integumentary	Hesperiden + Rosemary ^C^	Increased the minimal erythema dose after UV irradiation (51)
	Vitamin C + Vitamin E ^C^	Increased the minimal erythema dose after UV irradiation (52)
	Lycopene + Beta-carotene ^C^	Prevent erythema following exposure to UV irradiation (53)

S, synergistic study design; C, combination study design; EEG, electroencephalogram; CRP, C-reactive protein; T3, triiodothyronine; T4, thyroxine; TSH, thyroid stimulating hormone; TNF- α, tumor necrosis factor alpha; NAFLD, non-alcoholic fatty liver disease; UV, ultra-violet.

### Nutrient synergy and cardiovascular health

Nutrient synergy is particularly relevant in the context of cardiovascular health, where various nutrients play crucial roles in supporting heart health, blood flow, and vasculature compliance. Epidemiological data have consistently indicated that elevated levels of homocysteine in circulation are associated with an increased risk of cardiovascular disease (56, 57). Studies have shown that consuming adequate amounts of folate, vitamin B6, and vitamin B12 can work synergistically to lower homocysteine levels, thus reducing the risk of heart disease (58, 59). Specifically, data indicate that combining vitamin B12 with folic acid supplements optimizes the reduction in homocysteine levels in a study including 150 young women, potentially amplifying the advantages of these interventions in preventing cardiovascular disease (36). Additional work has been done examining the effects of CoQ10 and/or vitamin E on cardiometabolic outcomes in patients with polycystic ovary syndrome (PCOS), which is the most prevalent endocrine disorder in reproductive age women (37). Eighty-six women with PCOS were allocated to supplement with either CoQ10, vitamin E (as d-α -tocopherol), CoQ10 plus vitamin E, or placebo for 8 weeks. Interestingly, only the CoQ10 plus vitamin E treatment significantly reduced low-density lipoprotein cholesterol (LDL-C), increased high-density lipoprotein cholesterol (HDL-C), reduced atherogenic coefficient, and decreased visceral adiposity index values. Savinova et al. (38) conducted a parallel clinical trial assessing the effects of 2 g/day of extended release niacin, 4 g/day of omega-3 fatty acids, a combination of the two, or a respective dual placebo for 16 weeks on plasma lipids and lipoproteins in 56 adults with metabolic syndrome. The combination of niacin and omega-3 fatty acids demonstrated a synergistic effect, significantly increasing LDL apoE/apoB ratios and LDL apoA1/apoB ratios, suggesting that the enhanced cardiovascular effect likely arises from the combination therapy.

### Nutrient synergy and respiratory health

The adverse impact of pollution on various chronic respiratory conditions is well-documented (60–62). According to the World Health Organization, air pollution stands out as the most significant environmental health risk globally (63). Recent attention has been drawn to the potential of dietary changes and antioxidant supplementation in reducing the harmful effects of pollution in healthy populations and in individuals with conditions like asthma and other chronic respiratory diseases (64). Further, nutrient synergy may be an advantageous strategy as there are mixed data regarding supplementation with single vitamins for respiratory health (65, 66). Indeed, most studies which observed a benefit of antioxidant supplementation on protection against environmental pollutants involve both vitamin A and C (64). Likely due to a multi-targeted approach where, *in vitro,* vitamin C serves as a potent free radical scavenger, while vitamin A protects against membrane damage due to its ability to disrupt lipid peroxidation (67, 68). Grievink and colleagues (39) reported that daily supplementation of 500 mg of vitamin C and 100 mg of vitamin E for 15 weeks provided protection against the acute effects of ozone environmental pollution on forced expiratory volume (FEV) and forced vital capacity (FVC) in 38 non-smoking Danish cyclists. Additionally, an antioxidant intervention (650 mg vitamin C + 75 mg vitamin E + 15 mg beta-carotene) significantly improved FVC, FEV, and forced expiratory flow compared to a placebo in Mexican street workers exposed to hazardous environmental air conditions in a cross-over fashion. In follow-up assessments, it was also observed that the synergistic effects of the antioxidants provided a residual protective effect (40). Given that a majority of studies observing beneficial effects of antioxidant supplementation on respiratory function include a mixture of multiple nutrients, it is likely that the concept of nutrient synergy is driving these outcomes, possibly by an increase in total antioxidant capacity. However, a recurring limitation in understanding the synergistic potential of nutrients is the general lack of individual nutrient arms in many clinical trials utilizing nutrient combinations.

### Nutrient synergy and digestive health

One prime example of nutrient synergy specific to the gut microbiome and gut health is synbiotics. Synbiotics, in its name, is the synergy between prebiotics and probiotics. The intended purpose of a synbiotic is to deliver both prebiotics as well as probiotics to the human gut to exert health benefits. The prebiotic used can either directly support the efficacy of the probiotic itself or it can support the resident microbiota. For example, fructans can be very supportive of probiotic lactic acid bacteria and stimulate their growth (69), and glucose derived oligosaccharides like polydextrose can increase intestinal *Ruminococcus intestinalis* and enhance butyrate production (70). These are just a few of many in an exhaustive list that is beyond the scope of this review. However, through a strategic approach, unique prebiotics and probiotic species can be selected to exert specific and beneficial synergistic effects to the gut microbiome and ultimately to the host’s health. In a human clinical trial, Fujimori et al. (41) compared the effectiveness of probiotic (2 ×10^9^ CFU of *Bifidobacterium longum*), prebiotic (8 g psyllium), and synbiotic therapies (probiotic + prebiotic) in the treatment of ulcerative colitis using Inflammatory Bowel Disease Questionnaires and blood analysis. The results indicated that synbiotic therapy led to greater quality-of-life improvements and a significant decrease in C-reactive protein levels compared to probiotic or prebiotic treatment, suggesting its potential synergistic effect in treating ulcerative colitis. Additionally, one study found that a synbiotic consisting of *Lactobacillus acidophilus* (4×10^9^ CFU) with the prebiotic inulin (800 mg) was more effective in eradicating *Helicobacter pylori* when combined with a standard medication therapy as opposed to the medication regimen alone (71).

Beyond just the common example of synbiotics, there could be beneficial synergy between specific components of whole foods, phytonutrients, and microbes (both probiotics and resident microbiota). There is growing evidence that some phytonutrients (e.g., phenolic acids or flavonoids) can exert a prebiotic-like effect on the resident microbiota (42, 72–74). Despite this growing evidence, more data is needed to understand which specific phytonutrients or classes of phytonutrients provide a prebiotic effect and if these nutrients have unique interactions with particular probiotic species or strains. Therefore, more research is needed to fully understand the effects of using phytonutrients as a prebiotic in a synbiotic formulation.

### Nutrient synergy and musculoskeletal health

Bone health research has primarily focused on dietary intake or supplementation of vitamin D and calcium (Ca) due to their relationship with bone mass and fall risk, but several other micronutrients play a role in bone health [e.g., vitamin K, Mg, potassium (K)] (75). Although the data are mixed with some studies demonstrating no effect of supplemental vitamin D and/or Ca on bone health (76, 77) there are several well-controlled clinical trials and meta-analyses suggesting there is a synergistic relationship between intake of Ca and vitamins D and K on markers of bone health (i.e., bone mineral density, circulating levels of biomarkers associated with bone health, and risks of fractures) (44–47, 78). Two systematic reviews assessing the combined effects of vitamin D and Ca on fractures demonstrated significant reduction in fracture risk in middle-aged to older populations and osteoporotic individuals (46, 78). Furthermore, a clinical trial assessing the effects of vitamin D + Ca + vitamin K vs. vitamin D + Ca in postmenopausal women (>60 years) for 6-months demonstrated significantly better improvements in lumbar bone mineral density for the vitamin K group compared to no vitamin K (45). This may be partially explained by data indicating that vitamin D augments vitamin K-dependent bone proteins and triggers bone formation *in vitro* by upregulating expression of genes specific to osteoblasts (79, 80). Lastly, Gariballa et al. (47) demonstrated that vitamin D + Ca reduced parathyroid hormone and increased Ca/creatinine ratio after 6-months compared to vitamin D, Ca, or placebo in adults with relatively low baseline 25(OH)D levels (19.0–25.4 ng/mL). The results from Gariballa et al. (47) suggest that supplementation with calcium and vitamin D, compared to vitamin D or Ca individually, may improve bone health due to the relationship with high parathyroid hormone and increases in bone turnover (81). Taken together, the data from meta-analyses and clinical trials suggest there may be a synergistic effect of nutrients that are individually important for bone health.

The food matrix of whole food protein sources (e.g., milk, eggs) offer distinct nutrient compositions that can impact their effects on muscle protein synthesis (MPS) (82, 83). For example, protein sources which are high in micronutrients such as vitamin B12, zinc, choline, and selenium (e.g., whole eggs) appear to better support muscle growth and repair compared to protein sources lower in micronutrients (e.g., egg whites) indicating a synergistic relationship. Elliot et al. (84) was the first to demonstrate a more robust increase in MPS when comparing consumption of whole milk to skim milk following resistance exercise. Other studies have shown that acute consumption of whole eggs compared to the egg white alone leads to a more pronounced upregulation of mTORC1 and MPS response following resistance exercise (83, 85). A recent human trial also observed greater gains in muscle hypertrophy when adults consumed whole eggs following resistance training bouts for 12 weeks, compared to those which only consumed egg whites (86). Collectively, these data support a synergistic effect of the food matrix that increases the utilization of amino acids for muscle protein synthesis.

### Nutrient synergy and endocrine health

Multiple nutrients including selenium and zinc are necessary for hormone function, in particular thyroid hormones. Selenium is an essential cofactor for thyroid function as it is involved in thyroid hormone metabolizing enzymes known as selenoproteins. Selenium also works to protect the thyroid gland from oxidative stress (87, 88). Two large epidemiological studies found an inverse correlation between selenium levels and thyroid function metrics in mildly iodine deficient women (89, 90). Additionally, zinc plays a key role in the metabolism and function of various thyroid hormones, including thyroid-stimulating hormone (TSH) (43). Selenium and zinc together appear to have a synergistic benefit on biologically active forms of thyroid hormone. One double-blind randomized controlled clinical trial allocated women with obesity and hypothyroidism to 4 groups (30 mg zinc +200mcg selenium, 30 mg zinc, 200ug selenium, or placebo). The combination of selenium and zinc significantly improved free triiodothyronine (T3) compared to selenium alone or placebo. Further, only the zinc + selenium group significantly improved free thyroxine (T4) and TSH levels, with all three other groups eliciting no effect (43). Hill et al. (44) assessed the effects of a vitamin D, calcium, and leucine-enriched whey protein drink in 380 adults with sarcopenia for 13 weeks and reported suppression of parathyroid hormone, increased serum 25(OH)D, and accompanying small improvements in bone mineral density compared to an isocaloric control. Given the complex nature of endocrine function, more data is needed regarding the effects of synergistic nutrient combinations on male and female endocrine parameters.

### Nutrient synergy and immune function

Certain nutrients, when combined, can have more potent effects on immune response and overall immune health. A review of two double-blind randomized placebo-controlled trials suggests that there may be a synergistic benefit when vitamin C is combined with zinc for symptom relief from the common cold (49). A single-blinded human trial assessing the combination of vitamin C (1 g) and vitamin E (400 mg) in healthy adults showed the combination of the two vitamins improved Interlukin-1β and tumor necrosis factor-α (TNF-α) levels and reduced lipopolysaccharide-induced prostaglandin E2 production, suggesting an enhanced immune response (50). Another study evaluated the additive effects of *Chlorella vulgaris* (1.2 g) supplementation along with vitamin E (400 mg) in adults with obesity and non-alcoholic fatty liver disease (48). The study demonstrated significant improvements in bodyweight, fasting serum glucose, and TNF-α levels in the *Chlorella vulgaris* + vitamin E group compared to the placebo + vitamin E group, suggesting that *Chlorella vulgaris* could be an adjunctive therapy to improve weight management, inflammation, glucose control, and liver function in patients with non-alcoholic fatty liver disease.

### Nutrient synergy and integumentary health

Polyphenols and antioxidants are both bioactive compounds that, when used in combination, can provide synergistic effects that have been studied for their potential to offer mild protection against harmful ultraviolet (UV) radiation from the sun, retain moisture, and enhance the skin barrier function (91–93). When UV rays are absorbed by the skin, they can increase production of free radicals which can directly damage intracellular components such as DNA, proteins, lipids, and increase the risk of certain skin disorders (94). Vitamin E is a fat-soluble antioxidant primarily residing in cell membranes, where it protects the cell against lipid peroxidation. When vitamin E neutralizes a free radical, it becomes oxidized itself. However, *in vitro*, vitamin C can regenerate vitamin E by donating an electron to the oxidized vitamin E molecule, essentially recycling it and allowing it to continue its antioxidant function (95). In a double-blind placebo-controlled study, participants consumed vitamin C combined with vitamin E for 8 days leading up to a sunburn exposure to determine the minimal erythemal dose (MED) or the threshold in which a sunburn would occur (52). Data revealed that the vitamin C and vitamin E supplementation significantly increased the MED and reduced subcutaneous blood flow, indicating a protective effect against UV radiation. Lycopene (16 mg) and β-carotene (500mcg), two efficient singlet oxygen quenchers, have been shown to prevent erythema following exposure to UV irradiation compared to a placebo following 10 weeks of supplementation (53). Furthermore, a synergistic oral mixture of hesperidin, a type of citrus bioflavonoid, and rosemary for 12 weeks significantly increased the MED after UV irradiation at 8 and 12 weeks compared to placebo (51). This study also reported an *in vitro* experiment which showed that the hesperidin/rosemary combination allowed for a greater number of human keratinocytes surviving following UVB exposure while protecting against oxidative stress (51).

### Challenges investigating nutrient synergy and future directions

While nutrient synergy may be an accepted premise in the field of nutrition, there are relatively few studies which specifically investigate this phenomenon outside of whole food investigations. In recent decades, whole food interventions have been the main avenue of research investigating multiple nutrients in combination, in large part due to commodity grants (96). While this is promising for advancing our knowledge on the benefits of nutrient synergy, these studies cannot indicate which constituents of the whole food are driving the health benefit. To provide better solutions for human health, research is needed to identify which constituents of whole foods (e.g., vitamins, minerals, phytonutrients) are primarily responsible for the synergistic effects seen in the host. Furthermore, foods vary in nutrient content based on region or soil quality, and research shows that nutrient quality has declined over the decades (97, 98). Another limitation in interpreting some food interventions (via powder, juice, or whole food) is that the phytonutrient(s) responsible for a desired health outcome has not been quantified in methodology, making it difficult to determine if a Type II error occurred due to low levels of a desired constituent of the food (99–101).

It is also important to note that although there are some well-designed clinical trials ideally suited to investigate nutrient synergy (e.g., placebo, treatment A, treatment B, vs. treatment A + B), some of the studies included in this review are not properly designed to delineate a synergistic effect (e.g., treatment A + B vs. placebo) but a combination study design (see Table 1). Specifically, multivitamin and mineral research is especially challenging to elucidate nutrient synergy due to the large number of ingredients contained in the active treatments group compared only to a placebo. However, the reason these trials did not employ a true nutrient synergy design was likely in order to build upon previous evidence indicating these individual nutrients provided a benefit for the same endpoint or had a similar mechanism. These studies utilized the theoretical framework that the combination of nutrients will have a greater effect than the individual nutrients. Clinical trials specifically designed to assess nutrient synergy are also more expensive and require larger sample sizes compared to the trial designs employed in some studies discussed in this review.

Nutrients present in whole foods may have different effects on our health compared to nutrients that are supplemented in sufficient or even substantially higher amounts. Few nutrition experts would refute a “food first” approach to optimizing human health; it is also undeniable that consuming adequate amounts of certain nutrients to exert a physiological effect is often not practical or possible from whole food sources (102). Conversely, if the desire is to provide supplemental nutrients in the quantities they are provided in nature, researchers and companies can apply innovative delivery systems that combine different nutrients to further enhance bioavailability and absorption. For instance, encapsulating two or more synergistic nutrients in liposomes or nanoparticles may improve their uptake and distribution in the body, potentially enhancing their beneficial effects (103–105). Additionally, several studies have demonstrated superior effects of nutrient combinations at lower doses compared to larger amounts of the isolated nutrients and improved research practices may allow for more effective dosing strategies within the framework of synergism (106–108).

One key limitation in the current understanding of nutrient synergy is the lack of precise mechanisms of action. Many of the aforementioned studies examined key physiological outcomes that benefit from nutrient synergy, however the mechanism of how they are achieved is not always clear. Investigators may start utilizing cell culture models or new technologies to study the synergistic effects of different nutrients (109). For example, immune cells can be exposed to combinations of vitamins, minerals, and phytochemicals to exert specific changes in immune cell activity, cytokine production, or cell proliferation. Additionally, other investigators have suggested utilizing *in vitro* diffusion assays, checkerboard arrays, or time-kill assays to study the interactions between nutrients which are traditionally utilized in microbiology (110). Beyond cell models, utilizing an organ-on-a-chip model, which is a microfluidic cell culture device that replicates the structure and function of a specific human organ or tissue, may allow researchers to study organ-level responses to drugs, phytonutrients, or synergistic combinations of nutrients in a controlled environment (111–113). These models can provide mechanistic insights into how nutrients work together to support health and longevity.

Advances in technology and the new -omics era may help elucidate nutrient mechanisms of action and provide more nuance in synergy in clinical settings. Transcriptomics, proteomics, and metabolomics may clarify if unique nutrient combinations impact the ADME of specific nutrients. Advances in the field of microbiomics could also shed light on how the microbiome and their metabolites shape how nutrients are processed in the gastrointestinal tract, before even reaching systemic circulation. The application of nutrigenomics, or the understanding of unique polymorphisms in a person’s genome impacts metabolism, can help identify individuals who may need more or less of specific nutrients to achieve similar serum concentrations relative to a majority population. Ultimately, the use of all these -omics can be applied to demonstrate key differences in the way whole foods or unique nutrient combinations impact the body when compared to the individual nutrients alone.

Through the use of artificial intelligence (AI), it may be less daunting for researchers to utilize these emerging tools to gain a deeper understanding of the complex interactions between nutrients and biological systems (114). Leveraging this technology may allow for novel nutrient synergies to be discovered in a cost-efficient manner that can be harnessed to enhance health, prevent diseases, and optimize nutrition interventions (115, 116). AI algorithms can efficiently integrate data from various sources, especially sources with exhaustively large data sets (e.g., microbiome genomics, proteomics, metabolomics, clinical study outcomes), to create comprehensive analyses. These data sets can be analyzed to identify patterns and correlations between different nutrients and their effects on biological processes (117). For example, some researchers have already developed a “Combination Index” which uses mathematical modeling to quantitatively express the interaction between nutrients and the software which analyzed these processes may be facilitated and enhanced by the use of AI (118, 119). Despite this potential, there would still be a vital need for AI-driven nutrient synergy discoveries and its effects on human health to be validated by traditional experimental research and clinical studies (120).

While outside the purpose and scope of this review, it is important to note that alongside nutrient synergy exists the concept of nutrient antagonism (110, 118). Antagonism in nutrient synergy refers to a situation where the presence of one nutrient interferes with the absorption, utilization, excretion, or function of another nutrient (118, 119). More recently, the term “anti-nutrients” have been applied to perpetrator nutrients which exert an antagonistic effect on other nutrients (119). Common examples of these perpetrator nutrients include, but are not limited to phytic acid, lectins, oxalates, and tannins. Phytic acid, as an example, impedes the absorption of iron, zinc, magnesium, calcium, and manganese by forming insoluble salts with the ionized minerals (121, 122). A similar mechanism of action is true for oxalates which form insoluble salts with calcium and magnesium and tannins which form insoluble salts with iron (123, 124). Alternatively, lectins have been known to damage the brush border of proximal small intestine epithelial cells leading to impaired absorption by altering the permeability of the cells (125). Beyond nutrient-nutrient interactions, it is also important to understand that prescription medications can act as perpetrators and impede nutrient absorption. Several antibiotics (e.g., penicillins, fluoroquinolones, tetracyclines) can either inhibit the synthesis of vitamins, decrease absorption, or form insoluble complexes decreasing the bioavailability of various minerals and vitamins (126). Beyond antibiotics, antacids, proton pump inhibitors, metformin, antipsychotics, antiepileptics, ACE inhibitors, and aspirin are just a few in an exhaustive list of pharmacologic agents which have an equally diverse range of mechanisms which alter nutrient bioavailability (126). Antagonism can lead to reduced overall health benefits when certain nutrients are consumed together or consumed together in incorrect quantities and/or ratios. This highlights the complexity of nutrient relationships and emphasizes the need to carefully consider adopting improved research techniques and methodologies in the study of nutrient interactions.

## Conclusion

In conclusion, mounting evidence suggests that certain nutrients, when consumed together, can have a greater efficacy than when consumed alone and have a profound impact on health and longevity. Understanding the intricate mechanisms of nutrient synergy has implications for developing dietary strategies to support human health and potentially improve disease prevention. As research in this area continues to evolve, uncovering the full extent of nutrient synergy’s influence on health could pave the way for more targeted and effective interventions to promote overall well-being.

## Author contributions

JT: Conceptualization, Writing – original draft, Writing – review & editing. TK: Conceptualization, Writing – original draft, Writing – review & editing. PS: Conceptualization, Writing – original draft, Writing – review & editing. AG: Conceptualization, Writing – review & editing. TM: Conceptualization, Writing – review & editing. RE: Conceptualization, Writing – review & editing.
